# Individuals in food webs: the relationships between trophic position, omnivory and among-individual diet variation

**DOI:** 10.1007/s00442-014-3203-4

**Published:** 2015-02-05

**Authors:** Richard Svanbäck, Mario Quevedo, Jens Olsson, Peter Eklöv

**Affiliations:** 1 Department of Ecology and Genetics/Limnology, Uppsala University, Norbyvägen 18D, 752 36 Uppsala, Sweden; 2 Research Unit of Biodiversity (UO-PA-CSIC), University of Oviedo, Campus de Mieres, 33600 Mieres, Spain; 3 Department of Aquatic Resources, Institute of Coastal Research, Swedish University of Agricultural Sciences, Skolgatan 6, 74242 Öregrund, Sweden

**Keywords:** Trophic position, Evolution, Communities, Populations, Eco-evolutionary feedback

## Abstract

Among-individual diet variation is common in natural populations and may occur at any trophic level within a food web. Yet, little is known about its variation among trophic levels and how such variation could affect phenotypic divergence within populations. In this study we investigate the relationships between trophic position (the population’s range and average) and among-individual diet variation. We test for diet variation among individuals and across size classes of Eurasian perch (*Perca fluviatilis*), a widespread predatory freshwater fish that undergoes ontogenetic niche shifts. Second, we investigate among-individual diet variation within fish and invertebrate populations in two different lake communities using stable isotopes. Third, we test potential evolutionary implications of population trophic position by assessing the relationship between the proportion of piscivorous perch (populations of higher trophic position) and the degree of phenotypic divergence between littoral and pelagic perch sub-populations. We show that among-individual diet variation is highest at intermediate trophic positions, and that this high degree of among-individual variation likely causes an increase in the range of trophic positions among individuals. We also found that phenotypic divergence was negatively related to trophic position in a population. This study thus shows that trophic position is related to and may be important for among-individual diet variation as well as to phenotypic divergence within populations.

**Electronic supplementary material:**

The online version of this article (doi:10.1007/s00442-014-3203-4) contains supplementary material, which is available to authorized users.

## Introduction

Among-individual diet variation is common in natural populations and may occur at any trophic level within a food web (Bolnick et al. 2003), and could be important for both ecological and evolutionary processes (Bolnick et al. 2011, 2003; Quevedo et al. 2009). Both competition for food and predation influence the degree of among-individual diet variation (Eklöv and Svanbäck 2006; Svanbäck and Bolnick 2007; Svanbäck and Persson 2004). Intraspecific competition for food will increase the degree of among-individual diet variation (Svanbäck and Bolnick 2007; Svanbäck and Persson 2004; Svanbäck et al. 2011), whereas the risk of predation may decrease among-individual diet variation as a consequence of restricted habitat choice in prey (Eklöv and Svanbäck 2006). We know that the magnitude of among-individual diet variation varies widely among species, but we know much less regarding how this variation is related to the position of organisms in a food chain, i.e., to their trophic position.

The trophic position of organisms is a key aspect of their ecology. Species high up the food chain, e.g., top predators, can have a strong effect on the structure and dynamics of the community via predator–prey interactions (Casini et al. 2012; Menge 1997; Schmitz et al. 2000; Sih et al. 1985). Hairston et al. (1960) proposed that in tri-trophic food chains carnivores suppress herbivores indirectly allowing plants to grow unimpeded by predation (i.e., a trophic cascade). This indirect effect was later generalized by Oksanen et al. (1981) and found to be valid for systems of up to five trophic levels. According to the trophic cascade hypothesis, in a food chain of four trophic levels (i.e., plants–herbivores–intermediate predators–top predators), top predators and herbivores should be regulated by competition whereas intermediate predators should be regulated by predation (Hairston et al. 1960). Thus, if among-individual diet variation increases with intraspecific competition (Svanbäck and Bolnick 2007; Svanbäck and Persson 2004) and decreases with predation (Eklöv and Svanbäck 2006), we would expect top predators and herbivores in a four-trophic food chain to show higher among-individual diet variation than intermediate predators.

Omnivorous species exploit a wider range of resources, potentially increasing their among-individual diet variation. Although omnivory is of fundamental importance to our understanding of food web dynamics (Holt and Polis 1997; Vandermeer 2006), little is known about how species’ omnivory influences among-individual diet variation. Recent studies using stable isotopes have shown that omnivorous species are especially common at intermediate trophic positions (Jepsen and Winemiller 2002; Zhang et al. 2013). This suggests that among-individual diet variation may be more prevalent at intermediate trophic positions, contrasting with the previous prediction based on the trophic cascade theory, showing that the ecological implications of among-individual diet variation in food webs are still not well understood.

It has been shown that an increase in among-individual diet variation within a population leads to an increase in phenotypic variation and diversification (Eklöv and Svanbäck 2006). Hence, if trophic position influences among-individual diet variation, this might affect the degree of phenotypic diversification. Interestingly, a recent study has shown that piscivory (fish predators in aquatic systems) limits diversification of feeding morphology and speciation rates in centrarchid fishes (Collar et al. 2009). This suggests that among-individual diet variation might be reduced at high trophic levels, and that diversification would be limited at higher trophic positions in a community.

In this study we investigate whether among-individual diet variation varies systematically across trophic levels within species or within communities. We first investigate the relationship between trophic position and among-individual diet variation among size classes in a generalist species, the ontogenetic omnivore Eurasian perch (*Perca fluviatilis*; see Materials and methods for species’ description). Second, to test the generality of this relationship in lake communities we assess the relationships between among-individual diet variation, range of trophic position among individuals, and average trophic position in populations of fish and invertebrates in two different lake communities. Finally, we evaluate evolutionary implications of the relationship between the populations’ trophic position and among-individual diet variation by comparing the proportion of piscivorous perch in a population and the population’s degree of phenotypic divergence between littoral and pelagic perch. The proportion of piscivorous perch can vary among populations (Persson et al. 1991), and generally increase with average body size. Thus, if trophic position is related to among-individual diet variation, then perch is also a good model for testing potential evolutionary implications of among-individual diet variation.

## Materials and methods

### Species’ description

**Fig. 1:**
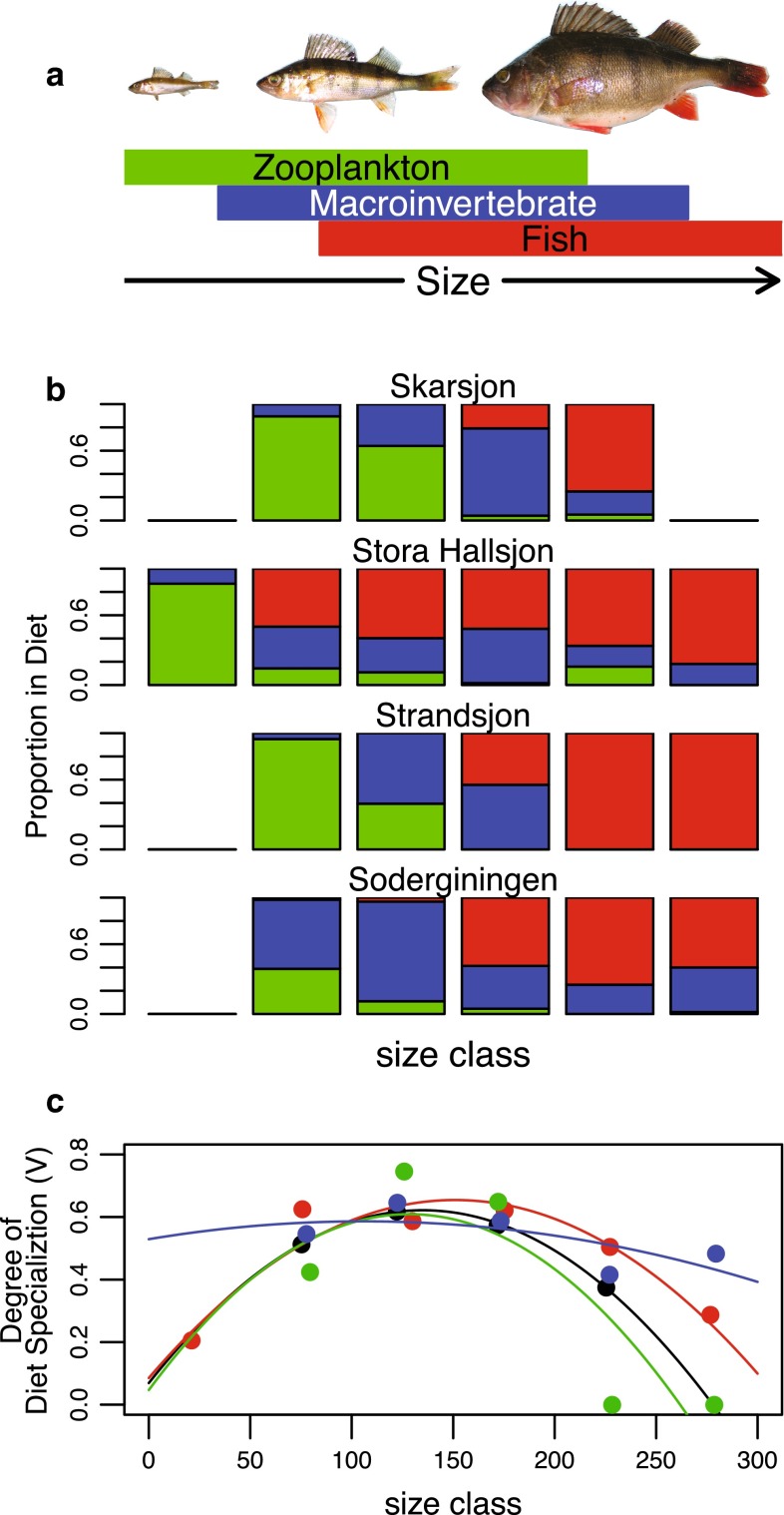
**a** A graphic presentation of diet for different size classes of perch based on previous work in Swedish lakes (Hjelm et al. 2000; Persson 1988; Svanbäck and Eklöv 2002). **b** The proportion of prey taxa in the diet of different size classes of perch in the four lakes studied: zooplankton (*green*), macroinvertebrates (*blue*) and fish (*red*). **c** Relationship between the degree of among-individual diet variation (*V*) based on stomach content analysis and perch size class in the four studied lakes. *Black symbols* and *black line* represent Lake Skärsjön, *red* *symbols* and *red line* represent Lake Stora Hållsjön, *green symbols* and *green line* represent Lake Strandsjön, and *blue symbols* and *blue line* represent Lake Söderginingen. Note that **b** and **c** shares the same* x*-axis. See Table S1 for sample sizes

Perch is considered a generalist species that may undergo two ontogenetic niche shifts during its life (Persson 1988). Juvenile Eurasian perch feed on zooplankton, then at intermediate sizes they shift to include macroinvertebrates in the diet. When large enough, they shift again to a diet mainly consisting of fish (Fig. 1a) (Hjelm et al. 2000; Persson 1988; Svanbäck and Eklöv 2002). Thus, as a perch gets larger it generally increases its trophic position, but it may also change the diet breadth over its ontogeny (Persson 1988; Quevedo et al. 2009; Svanbäck and Persson 2009).

Perch individuals in lakes specialize in feeding on either littoral or pelagic prey types (Quevedo et al. 2009; Svanbäck and Eklöv 2002; Svanbäck et al. 2008). This specialization is related to foraging efficiency trade-offs, where deeper bodied individuals are better foragers in the vegetated littoral habitat, whereas streamlined individuals are better foragers in the open-water pelagic habitat (Svanbäck and Eklöv 2003).

### Among-individual diet variation across size classes of perch

In mid-August 2001, we sampled perch in four lakes in the south-central part of Sweden (see Table 1 for lakes and numbers of perch sampled). In all lakes, perch were sampled both in the littoral and pelagic zones using multi-mesh gill nets of standard survey-link type. The littoral nets (30-m long and 1.5-m deep) and the pelagic nets (27.5-m long and 6-m deep) were set at 1500 and lifted at 0900 hours on the following day. The stomach contents of all fish were identified to the lowest possible taxonomic level, measured for length, converted to biomass (dry weight) and separated into seven different diet categories as described in Svanbäck and Persson (2004). Individual perch were then divided into 50-mm-increment size classes to enable calculation of diet variation among individuals in relation to size class.

**Table 1:** Lakes sampled for perch individual diet specialization and for community-wide diet variation; lake name, year sampled, coordinates, area, maximum depth and number of perch sampled are given

Lake	Year	Coordinates	Area (ha)	Maximum depth (m)	*n*
Lakes sampled for perch among-individual diet variation across size classes					
Söderginingen	2001	59º58′N, 18º16′E	303	2.8	124
Strandsjön	2001	59º52′N, 17º09′E	125	4	85
Stora Hållsjön	2001	60º00′N, 17º06′E	18	3.2	86
Skärsjön	2001	59º55′N, 18º01′E	31	9.1	119
Lakes samples for community-wide prevalence of diet variation in relation to trophic position					
Lötsjön	2004	59º52′N, 17º57′E	63	11.2	
Långsjön	2004	60º01′N, 17º34′E	250	12.5	

We quantified among-individual diet variation by first calculating mean diet overlap (i.e., mean proportional similarity) between each individual’s diet and the population diet ($\overline{PS}_{i}$) (Bolnick et al. 2002), where the population is defined as all individuals in one size class sampled. *PS*_*i*_ was calculated as *PS*_*i*_ = ∑ _*j*_min(*p*_*ij*_, *q*_*j*_), where *p*_*ij*_ is the frequency of diet category *j* in individual *i*’s diet, and *q*_*j*_ is the frequency of diet category *j* in the whole population (Bolnick et al. 2002). The degree of among-individual diet variation (*V*) was then calculated as $V = 1 - \overline{PS}_{i}$. This means that the degree of among-individual diet variation ranges from 0 when all individuals use the full range of resources used by the population, to 1 when individuals are more heterogeneous and use subsets of the resources used by the population.

Gut content may underestimate niche widths if there is limited diet information for each individual in the population, for example, if stomach size is small or resource competition constrains the number of prey consumed per individual. Therefore, to test whether the observed degree of *V* differs from random expectations, we ran a non-parametric Monte Carlo bootstrap simulation using 10,000 replicates (Bolnick et al. 2002). We generated null diet metrics drawn from the population (size-class) distribution from which the significance of the observed value was computed.

### Community-wide among-individual diet variation in relation to trophic position

In August and September 2004, we sampled the fish community in two lakes (Table 1). Littoral and pelagic nets (same as above) were set overnight. The use of multi-mesh gill nets of standard link type enabled us to catch fish of all size classes except the smallest size classes (<40 mm). This meant that we likely did not catch the full size range of all species as young-of-the-year fishes are less frequently caught in these nets. However, because we monitored these lakes for more than 10 years we are confident that we did not miss any small species that could have been present (e.g., minnows etc.). Benthic invertebrates were sampled using a hand net that was swept through the vegetation and over the bottom in the littoral zone. As we were only interested in a quantitative sample of benthic invertebrates from each lake we spent 1 day per lake sampling invertebrates. After sweeping through the vegetation and the bottom, samples were sieved through a 0.5-mm-mesh net to remove finer particles. The invertebrates were then picked alive in the field. Zooplankton was collected with a 100-μm-mesh net (diameter 25 cm) from the pelagic habitat in each lake. We dragged the zooplankton net behind a boat while rowing for about 20 min, and emptying the net several times. All samples were frozen immediately after collection. We used stable isotope ratios of carbon (δ^13^C) and nitrogen (δ^15^N) to discriminate between pelagic and littoral resource use, and to calculate the trophic position of the fish and invertebrates (France 1995; Fry 1988).

The isotopic values of primary producers are highly variable compared to those of consumers, thus we used tissues of primary consumers (snails and mussels, see below) to obtain integrated isotopic values of primary producers (isotopic endpoints) in each habitat (e.g., Vander Zanden and Rasmussen 1999). To obtain δ^13^C and δ^15^N endpoints for the littoral food chain, we collected pulmonated snails (*Lymnaea peregra*), which scrape algae from rocks and macrophytes. To obtain endpoints for the pelagic food chain we used filter-feeding zebra mussels (*Dreissena polymorpha*) in Lake Långsjön. These mussels were preferred over other filter feeders like zooplankters because their isotopic values reflect a longer period of integration of assimilated diet. However, zebra mussels were not available in lake Lötsjön; therefore we averaged the isotopic values of three zooplankton samples spread through the growing season (4 June, 6 July and 19 August 2004). Zooplankton was collected with a 100-µm-mesh net. This sampling scheme was intended to provide a period of integration of stable isotopes compatible with that of longer-lived consumers (Carleton and del Rio 2010). See Fig S1 in the Appendix for estimates of retention times of stable isotopes in muscle tissue of perch. Portions of muscle tissue were dissected from fish, snails and mussels.

We standardized the isotopic values of fish and invertebrates to allow cross-ecosystem comparisons (Newsome et al. 2007), namely estimating the trophic position and the relative contribution of the littoral food chain (littoral reliance) to isotopic values. We used a mixing model based on the empirical, pelagic and littoral isotopic endpoints (see above), and assumed enrichment values between trophic levels of +0.47 % for δ^13^C and +3.40 % for δ^15^N (Quevedo and Olsson 2006).

The trophic position of a species was calculated as the average trophic position of all individuals of that species. The range of trophic position among individuals within a species was calculated as the difference between the individual with the highest and lowest trophic position. The range of littoral-pelagic foraging in each species’ population was calculated as the difference between the individuals with the highest and lowest proportion of littoral reliance.

To calculate trophic niche width and structure for each species’ population we used quantitative metrics based on the position of individuals in the trophic position–littoral reliance plane (Layman et al. 2007; Newsome et al. 2007; Quevedo et al. 2009). We estimated the isotopic degree of among-individual diet variation by calculating the distance of each individual to the centroid of its species’ population in the trophic position–littoral reliance plane. Since this centroid distance metric is weighted by a central point of isotopic values, it is less sensitive than other metrics to the influence of extreme values (Brind’Amour and Dubois 2013).

### Relationship between trophic position (piscivory) and phenotypic divergence in perch

To investigate the relationship between the perch populations, average trophic position and the degree of phenotypic divergence between littoral and pelagic individuals we revisited a previous study by Olsson et al. (2006). Olsson et al. (2006) collected perch from the littoral and pelagic habitat using multi-mesh gill nets (same type as above) in 11 lakes in the Uppland region, Sweden. The littoral nets were set immediately outside the reed belt in the littoral zone and the pelagic nets were set at the surface in the central part of the lake. Morphology was analyzed using landmark-based morphometrics (Zelditch et al. 2004). Twenty-one landmarks were digitized on the left-hand side of each specimen. The morphology described by these landmarks is functionally related to foraging on littoral and pelagic prey types (Svanbäck and Eklöv 2003, 2004). We calculated the proportion of piscivorous perch based on the assumption that perch start to become piscivorous at the size of 120 mm and then linearly increase in their degree of piscivory until a size of 180 mm when they are fully piscivorous. Based on the diet results from the first part of this study, we know that perch may become piscivorous at smaller sizes (<120 mm) and also feed on macroinvertebrates at larger sizes (>180 mm), so we considered this index as a potential degree of piscivory in the perch population. Thus, we expect the degree of piscivory to be positively related to the average trophic position in a population. We then related the degree of piscivory in the population to the degree of phenotypic divergence between littoral and pelagic sampled perch. There was no evidence for differences in size between littoral and pelagic perch within each sampled lake (Olsson et al. 2006), implying that the morphological divergence between littoral and pelagic perch was size independent.

### Statistical analysis

We used general linear models (analysis of covariance) performed in the R statistical environment (R Development Core Team 2008) in all the parts of this study for identifying significant variables. To investigate how perch size class was related to the degree of *V*, we used lake as fixed factor and the median of the size class (Size) and Size^2^ as covariates. Similarly, to investigate how a species’ trophic position was related to isotopic among-individual diet variation, range of trophic position among individuals and range of littoral-pelagic foraging, we used lake as fixed factor and trophic position (TP) and TP^2^ as covariates. We included the quadratic terms (Size^2^ and TP^2^) to allow for intermediate levels of Size and TP to relate to among-individual diet variation differently than more extreme values of Size and TP. Furthermore, we used linear regression to investigate the relationship between trophic position (piscivory) and phenotypic divergence in perch populations. The significance level used in all tests was *P* < 0.05.

## Results

### Among-individual diet variation across size classes of perch

Perch diets over ontogeny largely confirmed the predictions. In general, perch in small size classes fed mostly on zooplankton, and included first macroinvertebrates and later fish with increasing size. At the largest size classes, perch were mainly piscivorous (Fig. 1b).

Among-individual diet variation in perch varied among size classes from *V* = 0 in size classes 200–250 mm and 250–300 mm in Lake Strandsjön to *V* = 0.75 in size class 100–150 mm in Lake Strandsjön (See Table S1 in the appendix). Overall, there was a strong quadratic effect of size on among-individual diet variation, which was highest at intermediate size classes (Fig. 1c; Table 2). There were no significant differences among lakes in the degree of among-individual diet variation in this study (Table 2). Monte Carlo simulations showed that the index of among-individual diet variation always differed from null expectations (*p* < 0.05), except for size classes 200–250 and 250–300 mm in Lake Strandsjön.

**Table 2:** Results from analyses of covariance (ANCOVAs) for the size-class diet specialization study testing for the effects of size and lake

	*df*	*F*-value	*P*-value
Size	1	0.003	0.957
Lake	3	1.392	0.314
Size^2^	1	16.164	0.004
Size × lake	3	1.945	0.201
Lake × size^2^	3	0.775	0.540
Residuals	8		

### Community-wide diet variation among individuals in relation to trophic position

**Fig. 2:**
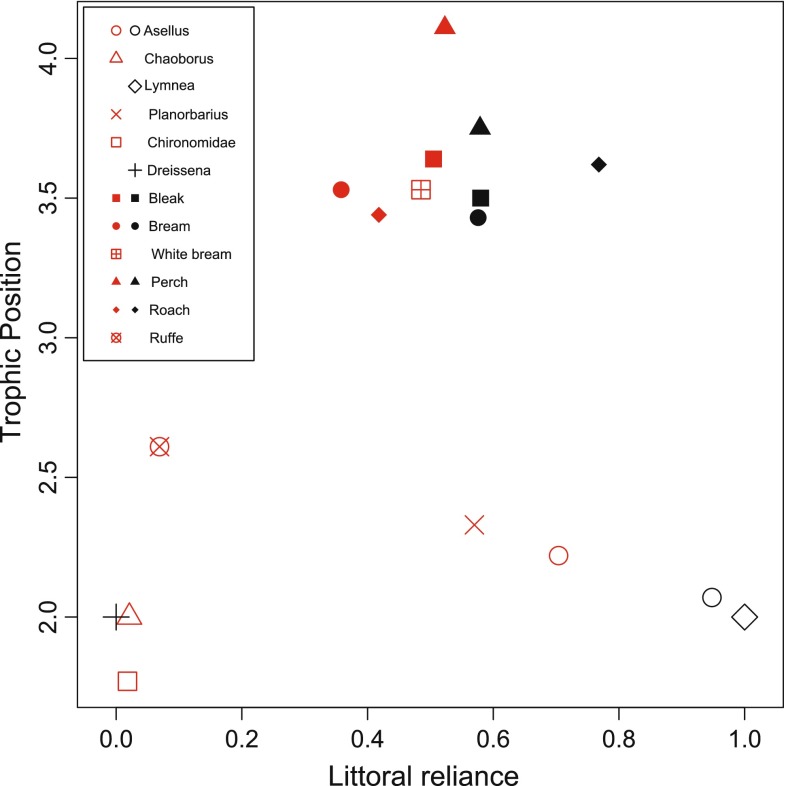
Organization of species in the trophic position–littoral reliance plane.* Littoral reliance* is the average proportion of carbon that comes from the littoral food chain, i.e., if littoral reliance is 0 then the population is feeding 100 % from the pelagic food chain whereas a littoral reliance of 1 means that the population is feeding 100 % from the littoral food chain. *Each dot* represents the average of a species from Lake Lötsjön (*red symbols*) and Lake Långsjön (*black symbols*). See Table S2 for sample sizes

**Fig. 3:**
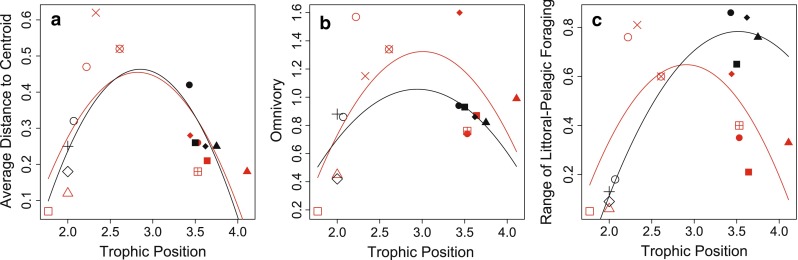
Relationship between average trophic position of a species and **a** the degree of among-individual diet variation calculated as average distance to the centroid ($\overline{CD}$), **b** degree of omnivory and **c** range of littoral-pelagic foraging. *Each dot* represents the value of a species (symbols are the same as in Fig. 2) from Lake Lötsjön (*red symbols*) and Lake Långsjön (*black symbols*). The *regression lines* are the best-fitting generalized linear model for each data set. See Table S1 for sample sizes

In both Lake Lötsjön and Lake Långsjön, lower trophic level species (invertebrates) tended to be on average either more littoral or more pelagic in their habitat use. Higher trophic level species (fish), on the other hand, tended to be more intermediate in using both littoral and pelagic food resources (Fig. 2; Table S2, appendix). When comparing among populations, trophic position showed a quadratic relationship in diet variation among individuals, measured as average distance to the centroid ($\overline{\text{CD}}$) of isotopic values; species at intermediate trophic positions had the highest degrees of among-individual diet variation (Fig. 3a; Table 3). There were no differences in this pattern between the lakes (Table 3).

**Table 3:** Results from ANCOVAS for the isotope study testing for the effects of trophic position (*TP*) and lake on isotopic diet specialization (average distance to centroid, $\overline{\text{CD}}$), degree of omnivory (range of trophic position) and range of littoral-pelagic foraging

	*df*	*F*-value	*P*-value
Distance to centroid			
TP	1	0.155	0.701
Lake	1	0.059	0.812
TP^2^	1	9.204	0.011
TP × lake	1	0.053	0.822
Lake × TP^2^	1	0.017	0.898
Residuals	11		
Range of trophic positions			
TP	1	1.125	0.312
Lake	1	0.758	0.403
TP^2^	1	4.932	0.048
TP × lake	1	0.287	0.603
Lake × TP^2^	1	0.040	0.847
Residuals	11		
Range of littoral-pelagic foraging			
TP	1	7.470	0.020
Lake	1	0.757	0.403
TP^2^	1	10.303	0.008
TP × lake	1	7.048	0.022
Lake × TP^2^	1	0.029	0.868
Residuals	11		

The range of trophic position among individuals as well as the range of littoral-pelagic niche use was also related to the average trophic position of any given population. The omnivory in the populations showed a quadratic relationship with the average trophic position, and we found no significant difference in this relationship between the two lakes (Fig. 3b; Table 3). Similarly, there was an overall quadratic relationship between the range of littoral-pelagic niche use in the species’ population and the species’ trophic position (Fig. 3c; Table 3). In the range of littoral-pelagic niche use we also found a positive relationship between the species’ trophic position and the range of littoral-pelagic foraging (Table 3).

### Relationship between trophic position (piscivory) and phenotypic divergence in perch

**Fig. 4:**
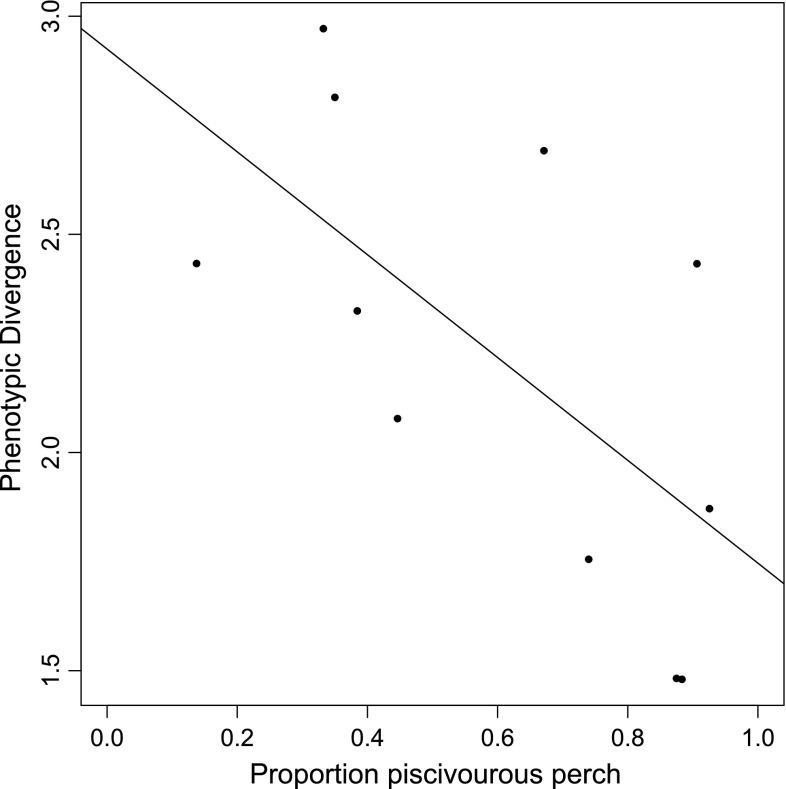
The relationship between the proportion of piscivorous perch and the phenotypic divergence between littoral and pelagic perch in the population. *Each dot* represents a lake and the *regression line* represents the relationship between the proportion of piscivorous perch in the population and the phenotypic divergence between littoral and pelagic perch in the population (*n* = 11, *R* ^2^ = 0.412, *P* = 0.033). The figure is based on data from Olsson et al. (2006)

We found a negative relationship between the proportion of piscivorous perch in the populations and the degree of divergence between perch sampled in the littoral and pelagic habitats (Fig. 4; *R* ^2^ = 0.412, *n* = 11, *P* = 0.033). A recent study using the same data and only focusing on the effect of intraspecific competition on perch divergence found that density of perch negatively affected divergence (Olsson et al. 2006). Adding both density of perch (catch per unit effort) and the proportion of piscivorous perch to the statistical model, however, lowered the statistical significance of the model (*R* ^2^ = 0.508, *P* = 0.058). In the extended model, neither the effect of density nor proportion of piscivorous fish had a significant effect on perch divergence. However, their effect sizes were both negative and of the same magnitude (density, *t* = −1.252, *P* = 0.246; proportion piscivores, *t* = −1.331, *P* = 0.220). Furthermore, we found a tenuous positive relationship between perch density and proportion of piscivores (*R* ^2^ = 0.284, *n* = 11, *P* = 0.053).

## Discussion

Among-individual diet variation is common in animal populations (Bolnick et al. 2003), and may be driven by intraspecific competition (Jones and Post 2013; Svanbäck and Bolnick 2007; Svanbäck and Persson 2004; Svanbäck et al. 2011), predation (Eklöv and Svanbäck 2006), or other interactions such as interspecific competition and intra-guild predation (Bolnick et al. 2010). Studying variation among individuals is important for understanding the ecology and evolution of populations (Bolnick et al. 2003, 2011). This is because population-level patterns are likely driven by individual-level phenomena (Araujo et al. 2011). Here we found that among-individual diet variation was highest at intermediate trophic levels. A wider range of trophic position among individuals at intermediate trophic levels supported the prediction that omnivory could determine patterns of among-individual diet variation in communities. The correlation between trophic level and the degree of phenotypic divergence in a population further suggests that trophic position in the population as a whole may have implications for morphological variation among individuals; phenotypic divergence between littoral and pelagic perch was lower in populations where the average individual had a higher trophic position.

How may consumer trophic position, omnivory and among-individual diet variation be related in a population? Optimal foraging theory predicts that the benefit of feeding on more than one prey type should be independent of the frequency of different resources, and exclusively dependent on the rarity of the most profitable resource (Stephens and Krebs 1986). However, functional trade-offs prevent any individual from using the whole spectrum of available resources (Bolnick et al. 2003; Skúlason and Smith 1995; Smith 1987). Such trade-offs can arise when individuals are better at using one resource type than others, potentially because prey consumption is tied to a certain morphology, physiology or cognitive trait (Estes et al. 2003; Lewis 1986; Persson 1985). We found a pattern of highest among-individual diet variation at intermediate trophic levels indicating that foraging of consumers at the intermediate trophic level may reflect shifts in cost–benefit foraging trade-offs.

**Fig. 5:**
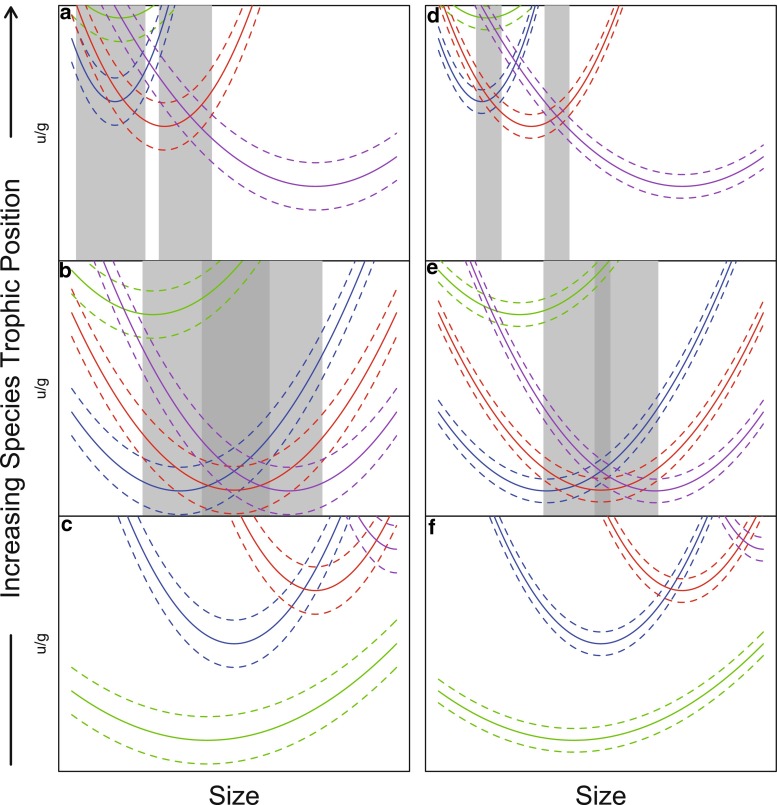
Hypothetical relationships between body size and the ratio of mortality (*μ*)/growth (*g*) expectancies of prey at different trophic positions. The figure shows a consumer’s “view” of the available prey environment. The conceptual model includes herbivores (**c, f**), intermediate carnivores (**b, e**) and top carnivores (**a, d**). *Green lines* indicate prey at the lowest trophic level (plants); then prey trophic level increases from *blue* through *red* and *purple* lines. The different types of resources are available to all consumers, yet using each resource type has a certain cost (*μ*) and benefit (*g*) ratio to the consumer expressed on the* vertical axis*, and this ratio varies for the consumer over its size range (*horizontal axis*). The different consumer types will have slightly different cost/benefit ratios at a specific size, reflected by* dashed lines* around an average (*solid line*) for each available resource. Scenarios of high **a–c **and low **d–f **variation among consumer types. Opportunities for among-individual diet variation by consumer types of different sizes are shown as* light-* *grey bars* (two resources available) and *dark bars* (three resources available). Any mechanism that causes consumer types to expand the variation in μ/g (interval width bounded by *dashed lines*) therefore increases the likelihood of among-individual diet variation of a consumer population by permitting alternate resources with equivalent fitness. One of the mechanisms that generates variants within a size class is trade-offs in foraging efficiencies, such as those observed across size classes (causing curvature in a resource function)

It is well know that consumers trade-off foraging gain versus mortality risk, especially when these vary with habitat (Werner and Gilliam 1984). This trade-off will change over the ontogeny of individuals, as well as across species, according to the balance of predation mortality risk and resource benefits. Such a trade-off can be depicted by the ratio of mortality to growth expectancy (Werner and Gilliam 1984). Central to this trade-off is that selection should favor foragers that minimize the ratio by either minimizing mortality or maximizing growth. How may such a trade-off affect the degree of among-individual diet variation and omnivory across trophic levels? In Fig. 5 we show a conceptual model of how variation among individuals in the mortality/growth expectancy ratio may vary with size, by means of trade-offs in foraging efficiencies on distinct resources. The relationship of the mortality/growth ratio with size shows distinct shapes both among consumer species as well as among individuals within a species because competition and predation pressures experienced by foragers vary over ontogeny. Such differences may arise as a consequence of foraging efficiency trade-offs discussed above, trade-offs in defense strategies (Chipps et al. 2004; Ruxton et al. 2004; Svanbäck and Eklöv 2011), and differences in animal personalities that will affect both foraging and predation risk (Mittelbach et al. 2014; Sih et al. 2004). For a predator at a relatively low trophic position that specializes on lower trophic level prey and thus trade-offs efficiency on higher trophic level prey this results in the consumption of few resource types, a low degree of omnivory and a low degree of among-individual diet variation (Fig. 5c, f). A predator at an intermediate trophic position should have a higher degree of omnivory both across different size classes and across different phenotypes within a size class, resulting in a higher degree of among-individual diet variation because foraging on different prey items should result in different foraging predation risk trade-offs (Fig. 5b, e). A top predator should have a lower degree of omnivory, and a low among-individual diet variation because in aquatic systems they are mostly piscivores, which should specialize on capturing large prey sizes relatively early in their ontogeny, trading off foraging gain to smaller prey sizes later in their ontogeny (Fig. 5a, d). However, we know little about how the trade-off between resource use and predation risk of foragers across trophic levels affects opportunities for among-individual diet variation. The model in Fig. 5 shows at least two uncertainties about among-individual diet variation. Firstly, among-individual diet variation will only be favored by selection under equivalent fitness for feeding on different resources (i.e., grey bars in Fig. 5a, d, b, e). Thus, the question will be how likely this occurs relative to non-equivalence (Fig. 5c, f). Secondly, crucial to such equivalency will be the variation within a consumer type (variation between dashed lines; Fig. 5) relative to the variation between resource types (variation between solid lines; Fig. 5). Less variation between resources in mortality/growth and more variation within a consumer type yield greater opportunity for fitness equivalence (see the contrast between Fig. 5a–c, d–f).

What are the causal relationships between the individual foraging strategy and phenotypic variation? As discussed above, trade-offs can regulate individual diet strategies and can arise through cognitive trade-offs to use multiple resources. For example, in perch it has been shown that foraging efficiency decreases when they try to exploit multiple compared to single prey types (Persson 1985). In this situation diet specialization would potentially lead to phenotypic variation. Trade-offs may also be mediated by size or by individual morphology. In that case, the causal relationship between individual diet variation and phenotypic variation would be reversed where phenotypic variation would lead to among-individual diet variation. Again, in perch there are trade-offs related to both phenotypic variation in body size and morphology (Byström and Garcia-Berthou 1999; Lundvall et al. 1999; Svanbäck and Eklöv 2003). In natural populations, both causal directions are possible. However the key uncertainty is whether one of the directions is more likely.

Phenotypic variation within a population arises as a result of differences in plastic developmental responses to resource use, but also through underlying genetic variation, or a combination of both (Stearns 1989, 1992). Numerous experiments on diet-induced phenotypic variation (Olsson et al. 2006; Robinson and Wilson 1996) as well as studies on genetic variation/differentiation in traits related to foraging (Rogers and Bernatchez 2007; Rogers et al. 2002; Schluter et al. 2010), have shown that both causal relations are acting in nature and can vary among populations (e.g., Parsons and Robinson 2006; Schlichting 2008; Svanbäck and Schluter 2012). Stable environments have been suggested to select for genetic variation (i.e., phenotypic variation would cause among-individual diet variation) whereas unstable, fluctuating environments would select for increased phenotypic plasticity (i.e., phenotypic variation would be a consequence of among-individual diet variation) (DeWitt and Scheiner 2004; Hori 1993; Scheiner 1993). However, to date, relatively little is known about the relative importance of plasticity and genetic variation for among-individual diet variation. The relative importance of phenotypic plasticity and genetic variation for among-individual diet variation would likely differ due to variation in competition and predation. In order to understand how competition and predation influence among-individual diet variation we would need to conduct a common garden experiment where the level of competition and/or predation is varied. Experiments would also have to be performed on populations with varying degrees of phenotypic determination from genetic variation and phenotypic plasticity. The experiments would ultimately tell us about the relative roles of extrinsic (e.g., competition and predation) and intrinsic (genetic variation and phenotypic plasticity) factors in influencing among-individual diet variation.

### Ecological and evolutionary implications of among-individual diet variation

The among-individual variation we found in the use of littoral and pelagic food webs suggests that individuals contribute distinctly to habitat coupling. Theoretical studies suggest that mobile generalist predators that connect separate food chains through predation may enhance food web stability (McCann et al. 2005; Rooney et al. 2006). However, the role of predators in linking food webs has both theoretically and empirically mostly been studied by treating populations as homogeneous entities, without considering potential effects of among-individual variation in the use of habitats and resources (but see e.g., Harrod et al. 2010; Hayden et al. 2013; Quevedo et al. 2009). By focusing on individual variation in the food web we may be able to better predict how species interact with each other. A number of theoretical studies have also shown that intraspecific trait variation (e.g., among-individual diet variation) can affect population dynamics (Doebeli 1996; Fox and Kendall 2002). However, these studies were based only on single species and little is known about how intraspecific trait variation within a whole community affects population and community stability. Our study shows that the community aspect of intraspecific trait variation for population and community stability may be of great importance but more empirical and theoretical research is needed.

The relationship between among-individual diet variation and the average trophic position in the population might have consequences for phenotypic divergence between subpopulations. This can even lead to eco-evolutionary feedbacks if the phenotypic divergence leads to differences in food web coupling between habitats (Quevedo et al. 2009). Eklöv and Svanbäck (2006) showed experimentally that morphological variation increased with increasing degree of among-individual diet variation. In this study we found a negative correlation between the degree of piscivory (trophic position) in perch populations and the degree of phenotypic divergence between littoral and pelagic perch. This correlation was not significant when intraspecific competition (as measured by density) was included in the model. However, the trend was in the expected direction based on decreased among-individual diet variation at higher trophic levels (see Fig. 1c). In corroboration with our findings in perch, Collar et al. (2009) showed that piscivory limits diversification of feeding morphology and speciation rates in centrarchid fishes. Whether the phenotypic divergence in our perch populations is a result of genetic divergence or phenotypic plasticity is unknown at present. The greater phenotypic divergence at intermediate trophic levels suggests that species at these positions may have a higher likelihood for eco-evolutionary dynamics to drive phenotypic divergence relative to other trophic levels in lake communities; however, more data are needed to verify this finding and its evolutionary importance.

In conclusion, we found relationships between trophic position (the population’s range and average) and among-individual diet variation. Predation and competition as regulating factors of different trophic levels in the classic theory of trophic cascade cannot explain the patterns that we found. Instead, the range of trophic position among individuals, probably related to foraging-predation risk trade-offs across trophic levels, seems likely to explain our patterns. However, both the mechanisms (trade-offs) behind the relationships and the causal relationships between these correlations need to be studied further, as well as the ecological and evolutionary implications of our findings.

#### **Author contribution statement**

RS conceived and designed the study, RS, MQ, JO and PE performed the research, RS analyzed the data, RS, MQ, JO, and PE wrote the paper.

## Electronic supplementary material

Below is the link to the electronic supplementary material.

Supplementary material 1 (DOCX 106 kb)
