# Supplementary material for: Individuals in food webs: the relationships between trophic position, omnivory and among-individual diet variation
Source: Oecologia. 2015 Feb 5;178(1):103–14. doi: 10.1007/s00442-014-3203-4 (PMC4555210; doi:10.1007/s00442-014-3203-4)
Supplement: Supplementary file 1 — Supplementary material 1 (DOCX 106 kb) [file 442_2014_3203_MOESM1_ESM.docx]

Electronic Supplementary Material

for

Individuals in food webs: the relationships between trophic position, omnivory and among-individual diet variation

by

Richard Svanbäck^1,*^, Mario Quevedo^2^, Jens Olsson^3^, and Peter Eklöv^1^

^1^Dept of Ecology and Genetics/Limnology, Uppsala University, Norbyvägen 18D, 752 36 Uppsala, Sweden

^2^ Research Unit of Biodiversity (UO-PA-CSIC), University of Oviedo, Campus de Mieres, E-33600 Mieres, Spain

^3^Institute of coastal research, Department of Aquatic Resources, Swedish University of Agricultural Sciences, Skolgatan 6, 74242 Öregrund, Sweden.

^*^Author for correspondence ([richard.svanback@ebc.uu.se](mailto:richard.svanback@ebc.uu.se))

Table S1. The table shows the degree of individual specialization (V), Standard deviation (SD) and the number of individuals (N) used from each size class in the four lakes.

Lake Size class V SD N

Skärsjön 51-100 0.513 0.203 19

Skärsjön 101-150 0.618 0.181 71

Skärsjön 151-200 0.574 0.225 24

Skärsjön 201-250 0.375 0.250 11

Stora Hållsjön 1-50 0.206 0.193 11

Stora Hållsjön 51-100 0.625 0.173 16

Stora Hållsjön 101-150 0.587 0.242 15

Stora Hållsjön 151-200 0.622 0.161 27

Stora Hållsjön 201-250 0.505 0.258 19

Stora Hållsjön 251-300 0.288 0.267 12

Strandsjön 51-100 0.424 0.225 35

Strandsjön 101-150 0.746 0.077 27

Strandsjön 151-200 0.649 0.105 9

Strandsjön 201-250 0 0 7

Strandsjön 251-300 0 0 7

Söderginingen 51-100 0.546 0.185 56

Söderginingen 101-150 0.646 0.154 29

Söderginingen 151-200 0.588 0.228 22

Söderginingen 201-250 0.416 0.301 24

Söderginingen 251-300 0.483 0.114 10

Table S2. Average and range (within parentheses) for values for trophic position (TP), and Littoral Reliance (LR), average and standard deviation (within parentheses) for distance to centroid (CD) and the number of individuals used (N) for each species in the two lakes examined.

Lake Species TP LR CD N

Långsjön Asellus 2.1 (0.86) 0.95 (0.18) 0.32 (0.19) 8

Långsjön Lymnea 1.0 (0.42) 1.0 (0.09) 0.18 (0.08) 7

Långsjön Dreissena 2.0 (0.88) 0.0 (0.13) 0.25 (0.18) 10

Långsjön Bleak 3.5 (0.93) 0.58 (0.65) 0.26 (0.14) 30

Långsjön Bream 3.4 (0.94) 0.58 (0.86) 0.42 (0.28) 8

Långsjön Perch 3.8 (0.82) 0.58 (0.76) 0.25 (0.12) 67

Långsjön Roach 3.6 (0.86) 0.77 (0.84) 0.25 (0.11) 52

Lötsjön Asellus 2.2 (1.57) 0.70 (0.76) 0.47 (0.35) 10

Lötsjön Chaoborus 2 .0 (0.45) 0.02 (0.06) 0.12 (0.10) 7

Lötsjön Planorbarius 2.3 (1.15) 0.57 (0.81) 0.62 (0.07) 8

Lötsjön Chironomidae 1.77 (0.19) 0.02 (0.05) 0.07 (0.05) 9

Lötsjön Bleak 3.6 (0.87) 0.51 (0.21) 0.21 (0.13) 25

Lötsjön Bream 3.5 (0.74) 0.36 (0.35) 0.26 (0.11) 11

Lötsjön White bream 3.53 (0.76) 0.49 (0.40) 0.18 (0.10) 32

Lötsjön Perch 4.1 (0.99) 0.52 (0.33) 0.18 (0.14) 76

Lötsjön Roach 3.4 (1.60) 0.42 (0.61) 0.28 (0.21) 84

Lötsjön Ruffe 2.6 (1.34) 0.07 (0.60) 0.52 (0.21) 8


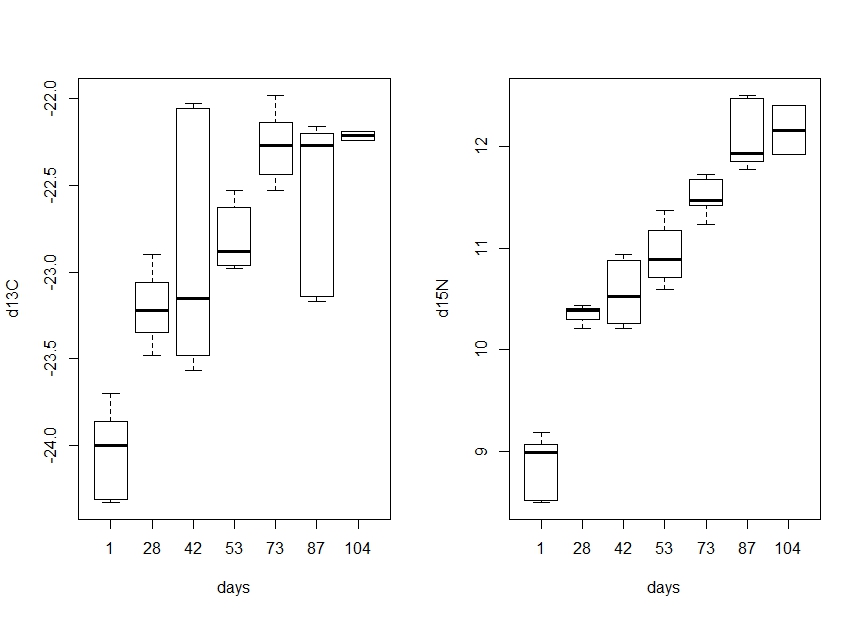


Figure S1. Time-course of values of stable isotopes ^13^C and ^15^N in muscle tissue of 0+ perch after diet switch. We fed the fish (about 5 g) *ad libitum* a constant diet of chironomid larvae, and kept them at 20° C. Average retention times were 48.5 (SE = 23; p = 0.044) and 117 (SE = 44, p = 0.011) days for ^13^C and ^15^N, respectively. Note that standard errors of the estimates were wide in both cases. We modelled retention times as one-compartment exponentials ([del Rio and Anderson-Sprecher 2008](#_ENREF_1))

**Supplementary references**

del Rio CM, Anderson-Sprecher R (2008) Beyond the reaction progress variable: the meaning and significance of isotopic incorporation data. Oecologia 156:765-772. doi: 10.1007/s00442-008-1040-z
